# Noninvasive scoring systems predict hepatic and extra-hepatic cancers in patients with nonalcoholic fatty liver disease

**DOI:** 10.1371/journal.pone.0202393

**Published:** 2018-08-14

**Authors:** Noam Peleg, Orly Sneh Arbib, Assaf Issachar, Michal Cohen-Naftaly, Marius Braun, Amir Shlomai

**Affiliations:** 1 Department of Medicine D, Rabin Medical Center, Beilinson hospital, Petach-Tikva, Israel; 2 The Liver Institute, Rabin Medical Center, Beilinson hospital, Petach-Tikva, Israel; 3 Sackler Faculty of Medicine, Tel Aviv University, Tel Aviv, Israel; Policlinico Universitario Campus Bio-Medico, ITALY

## Abstract

**Background:**

Liver fibrosis predicts liver-related morbidity and mortality in patients with non-alcoholic fatty liver disease (NAFLD). Non-invasive scores correlate with the degree of liver fibrosis in these patients.

**Aims and methods:**

To investigate the accuracy of noninvasive scoring systems in predicting long-term outcomes and cancer incidence of patients with NAFLD, we performed a single-center retrospective study of patients with biopsy proven NAFLD. Mean follow up period was 100 months. Outcomes included liver-related complications, hospitalizations, overall mortality and the development of any malignancies.

**Results:**

32 patients had advanced fibrosis (F3-F4) per biopsy at baseline and 121 patients had mild to moderate fibrosis (F0-F2). Both advanced histologic fibrosis stage as well as higher non-invasive scores predicted repeated hospitalizations and longer hospitalization stays. In a multivariate analysis, liver fibrosis (p = 0.002), FIB-4 score (p<0.001), NFS (p<0.001) but not APRI score (p = 0.07) were predictors of overall mortality, and the occurrence of malignancies was associated with higher APRI (p<0.001), FIB-4 (p<0.001) and NFS (p = 0.008) scores, but not with advanced fibrosis, as determined by liver biopsy (p = 0.105).

**Conclusions:**

In NAFLD patients, noninvasive scoring systems are good predictors of morbidity and mortality and may have an additive value in predicting the development of hepatic and extra-hepatic cancers.

## Introduction

Nonalcoholic fatty liver disease (NAFLD) is becoming a major cause of chronic liver disease and liver related morbidity and mortality worldwide [1, 2]. The most common risk factors for NAFLD are type 2 diabetes mellitus (DM) and insulin resistance, obesity and hypertension[3]. Advanced liver fibrosis stage is an important risk factor for progression to cirrhosis[4]. Most liver related outcomes occur once cirrhosis has developed, with the exception of hepatocellular carcinoma (HCC) that might develop even without cirrhosis in a proportion of NAFLD patients[5]. As a major etiology for end-stage liver disease, NAFLD is currently the second most common cause of HCC requiring liver transplantation[6, 7].

Percutaneous liver biopsy is considered the gold standard for assessment of the degree of liver fibrosis and inflammation. However, this procedure is associated with risk of serious complications and a considerable rate of sampling error and observer variations[8]. Several noninvasive scoring systems, composed of routinely measured clinical and laboratory variables, have been proposed to discriminate between patients with NAFLD with or without advanced liver fibrosis[9]. Limited data suggests that these noninvasive scoring systems can also be used to predict liver-related morbidity and mortality in NAFLD patients[10, 11]. Nonetheless, much less in known about the utility of these scores in predicting hepatic and extra-hepatic malignancies and their related co-morbidities in patients with long standing NAFLD. Given the evidence for increased frequency of some malignancies in patients with diabetes and the metabolic syndrome[12, 13], non-invasive predictors for common cancers in those patients are an unmet need.

Recently, we have shown the utility of the AST to platelets ratio index (APRI) score, FIB-4 score and the NAFLD fibrosis score (NFS) in predicting advanced fibrosis in patients with biopsy-proven NAFLD[14]. In the present study, we used this cohort of patients to further evaluate the accuracy of non-invasive scores in predicting long-term outcomes of patients with NAFLD. The parameters investigated here include all-cause mortality, liver-related complications, as well as the incidence of liver and non-liver related cancer.

## Patients & methods

### Study design

The study was approved by the local institutional review board (IRB), the Helsinki Committee of Rabin Medical Center, and all clinical investigations have been conducted according to the principles expressed in the Declaration of Helsinki. Due to its retrospective design, the ethics committee waived the requirement for informed consent for this study, and all data were fully anonymized. All documented liver biopsies performed from August 2005 to December 2012 and patients’ demographic, clinical and laboratory data, obtained from their electronic records, were reviewed, and only those with unequivocal diagnosis of NAFLD were included in the analysis. Other etiologies for chronic liver disease were excluded. Other etiologies for liver biopsy during the same time frame of our study were previously described [14]. The reasons for performing a liver biopsy in our cohort of patients were obtained from the electronic records. The reasons for liver biopsy were as follows: unclear diagnosis after clinical and laboratory evaluation (82 patients, 53.6%), unexplained splenomegaly (21 patients, 13.7%) and elevated ALT and suspected non-alcoholic steatohepatitis (NASH) (44 patients, 28.7%). 6 of the patients (3.9%) had no specific reason for liver biopsy cited in their electronic records.

Data regarding morbidity, mortality, hospitalizations, and cancer was obtained from the electronic records of the study population. Patients were assigned to have the diagnosis of diabetes mellitus in case of documented use of oral hypoglycemic drugs or insulin, or if the general practitioner has made this diagnosis according to established guidelines. Body Mass Index (BMI) was calculated using the formula: weight (in Kg)/height (in meters)^2^. APRI score, FIB-4 score and NFS were calculated using the original reported formulas[15]. High cut offs of these scoring systems were chosen for various analyses in this study, in order to provide high positive predictive values for advanced liver fibrosis[16, 17].

Histological examination was performed by an experienced pathologist. The degree of fibrosis was reported using the Metavir score as described previously [14], and the level of fatty infiltration was assessed and graded on a scale from 1 to 3 (1 = up to 30% of hepatocytes affected, 2 = 30%-60% of hepatocytes affected, 3 = more than 70% of hepatocytes affected). The presence of NASH was determined according to the scoring system of the NASH clinical research network[18].

### Statistical analysis

The primary end point of the study was to define the occurrence of all-cause mortality or the diagnosis of hepatic or extra hepatic malignancy during the follow up period. In addition, we aimed to define the incidence of liver-related complications such as the development of ascites, gastroesophageal varices, hepatic encephalopathy and Trans-jugular Intrahepatic Porto-systemic Shunt (TIPS) placement, as well as the number and length of hospitalizations during the follow up period. Statistical analysis was performed using the SAS 9.4 software. Categorical variables were compared by X^2^ or the Fisher exact tests, whereas continuous variables were compared with the Student’s t test. Correlation was evaluated by the Pearson correlation coefficient. A 2-sided P value of less than 0.05 was considered statistically significant. A stepwise logistic regression was made to identify independent factors associated with the study endpoint. Variables with missing values in more than 20% of the patients were not included in the regression analysis. The diagnostic accuracy of the three scoring systems (APRI, FIB-4 and NFS) to distinguish between patients with or without increased risk for the outcomes was investigated by determining the area under the receiver operating characteristic (ROC) curves. Cumulative liver-related events, overall mortality, diagnosis of cancer and liver transplantation were calculated using Kaplan–Meier analysis and compared by log-rank testing. Time at risk (T_0_) was considered as the time from the date of liver biopsy to the date of outcome or to the last day of follow up.

## Results

### Baseline characteristics

153 patients with biopsy proven NAFLD were enrolled to the study. Table 1 describes the baseline characteristics of the study population according to the degree of liver fibrosis, as determined by liver biopsy at baseline. 32 patients had advanced fibrosis (F3-F4) and 121 patients had no or mild to moderate fibrosis (F0-F2). The degree of liver fibrosis in the study population, as determined by liver biopsy, correlated well with the non-invasive scores (APRI, FIB-4 and NFS) that were significantly higher in patients with advanced fibrosis compared to non-advanced fibrosis, as previously described[14].

**Table 1:** Characteristics of study population according to liver fibrosis stage.

	Fibrosis 0–2 (N = 121)	Fibrosis 3–4 (N = 32)	p Value
**Gender–male%(N)**	54.55% (66)	59.38% (19)	0.69
**Age (range)**	47.7 (20–78)	56.09 (38–77)	<0.001
**BMI (range)**	29.4 (17.48–43.8)	30.06 (22–43.1)	0.48
**Statin use % (N)**	44.63% (54)	90.63% (29)	<0.001
**HTN diagnosis% (N)**	42.98% (52)	34.38% (11)	0.42
**DM2% (N)**	57.85% (70)	84.38% (27)	0.007
**Hemoglobin A1C (%) (range)**	6.05 (4.4–10.1)	7.11 (4.6–12)	0.010
**Total cholesterol (mg/dL) (range)**	185.56 (97–320.1)	156.06 (69–213)	<0.001
**Triglycerides (mg/dL) (range)**	169.16 (50–578)	131.55 (45–410)	0.04
**Creatinine (mg/dL) (range)**	0.80 (0.3–2.2)	0.74 (0.41–1.63)	0.24
**INR (range)**	1.06 (0.8–1.4)	1.16 (0.9–1.6)	<0.001
**Albumin (g/dL) (range)**	4.27 (2.1–5.3)	3.83 (1.9–4.8)	<0.001
**Platelets (range)**	242.98 (78–390)	134.84 (32–316)	<0.001
**AST (U/L) (range)**	54.78 (11–192)	64.72 (12–242)	0.20
**ALT (U/L) (range)**	76.60 (11–239)	63.09 (9–323)	0.18
**Presence of NASH % (N)**	14.87% (18)	28.12% (9)	0.12
**Steatosis grade**			0.16
**Steatosis grade 1% (N)**	37.19% (45)	43.75% (14)	
**Steatosis grade 2% (N)**	42.14% (51)	50.00% (16)	
**Steatosis grade 3% (N)**	20.66% (25)	6.25% (2)	
**APRI Score (range)**	0.83 (0.1–5.22)	2.04 (0.33–8.94)	0.001
**FIB-4 Score (range)**	1.44 (0.27–6.46)	5.30 (1.26–29.37)	<0.001
**NFS (range)**	-1.63 (-4.85–2.28)	1.21 (-3.78–4.7)	<0.001

NFS = NAFLD fibrosis score, HTN = hypertension, DM2 = type 2 diabetes mellitus, APRI = AST to Platelet Ratio Index, NASH = Nonalcoholic steatohepatitis.

Patients with advanced fibrosis were significantly older compared to patients with non-significant fibrosis (a mean age of 56.09 years vs. a mean age of 47.7 years, respectively, p<0.001). As expected, patients with advanced fibrosis had a higher INR and lower albumin and thrombocytes, reflecting the severity of their liver disease. Patients with advanced fibrosis were also more likely to have type 2 DM (84.38% vs. 57.85%, p = 0.007), with higher levels of hemoglobin A1C (7.11% vs. 6.05%, p = 0.01). Nonetheless, the two groups did not significantly differ regarding the grade of liver steatosis (p = 0.16), and although there was a trend for a higher incidence of NASH among patients with advanced fibrosis, the difference between the groups was not statistically significant (28.12% in patients with F3-F4 vs. 14.87% in patients with F0-F2, p = 0.12).

### Major clinical outcomes during patients’ follow-up

The mean documented follow-up of the 153 study participants was 100.23 months (range from 60.87 to 144.54 months). Table 2 outlines the major clinical outcomes during the follow-up period according to baseline liver fibrosis stage. During follow-up, patients with advanced fibrosis were more likely to be hospitalized with an average of 4.71 hospital admissions for patients with advanced fibrosis (STDV 5.01, median 3), compared with 1.66 hospital admissions for patients with mild to moderate fibrosis (STDV 2.9, median 0) (p = 0.003). The average hospitalization length was significantly longer in the group of patients with advanced fibrosis, as well (3.72 days compared with 1.99 days, p = 0.006). As expected, patients with advanced liver fibrosis were also more likely to have liver-related complications, such as ascites, encephalopathy and varices during the follow-up period. These patients also had an increased rate of all-cause mortality (34.37% compared to 6.61%, p<0.001). However, patients with advanced fibrosis per liver biopsy did not have more malignancies during follow up compared to patients without advanced liver fibrosis (p = 0.321).

**Table 2:** Major clinical outcomes of study population during the follow-up period according to liver fibrosis stage.

	Fibrosis 0–2 (N = 121)	Fibrosis 3–4 (N = 32)	p Value
**Length of follow up (months) (range)**	100.38 (60.87-144-54)	98.74 (61.8–140.7)	0.73
**Hospitalizations (No.) (range)**	1.66 (0–16)	4.71 (0–16)	0.003
**Length of hospitalizations (days) (range)**	1.99 (0–13)	3.72 (0–18)	0.006
**Ascites % (N)**	4.96% (6)	40.63% (13)	<0.001
**Encephalopathy % (N)**	3.31% (4)	28.13% (9)	<0.001
**Varices% (N)**	2.48% (3)	53.13% (17)	<0.001
**TIPS % (N)**	0.83% (1)	6.45% (2)	0.11
**Malignancy % (N)**	17.36% (12)	25% (8)	0.32
**All-cause mortality% (N)**	6.61% (8)	34.37% (11)	<0.001

TIPS = Trans-jugular Intrahepatic Portosystemic Shunt.

Table 3 presents the major clinical outcomes during the follow-up period according to the severity of fibrosis (advanced versus non-advanced fibrosis), as determined by three major noninvasive scoring systems. Higher baseline APRI, FIB-4 and NFS were associated with significantly higher all-cause mortality rates (26.92% versus 9.44%, p = 0.014 for APRI, 35.48% versus 6.55%, p<0.001 for FIB-4, 50% versus 5.42%, p<0.001 for NFS), higher admission rates (5.076 versus 1.704, p<0.001 for APRI, 5.833 versus 1.404, p<0.001 for FIB-4 and 6.26 versus 1.8, p<0.001 for NFS) and longer hospitalization stays (5.224 versus 1.748, p<0.001 for APRI, 4.929 versus 1.706, p<0.001 for FIB-4 score, 4.249 versus 2.308, p = 0.017 for NFS). In addition, NAFLD patients with higher scores were more likely to develop liver complications and had more malignancies during follow-up compared to patients with lower fibrosis scores (Table 3).

**Table 3:** Major clinical outcomes of study population during the follow-up period according to noninvasive scoring systems.

	APRI score			FIB-4 score			NFS		
	≤1.5 (127)	>1.5 (26)	p value	≤2.67 (122)	>2.67 (31)	p value	≤0.676 (105)	>0.676 (25)	p value
**Length of follow up (months)**	100.28	98.88	0.23	100.6	97.84	0.27	101.14	99.34	0.31
**Admissions (No.)**	1.70	5.07	<0.001	1.40	5.83	<0.001	1.8	6.26	<0.001
**Length of hospitalizations (days)**	1.74	5.22	<0.001	1.70	4.92	<0.001	2.308	4.24	0.01
**Ascites % (N)**	9.44% (12)	26.92% (7)	0.01	4.91% (6)	41.93% (13)	<0.001	3.1% (4)	62.5% (15)	<0.001
**Encephalopathy % (N)**	5.51% (7)	23.07% (6)	0.003	2.45% (3)	32.25% (10)	<0.001	2.32% (3)	41.6% (10)	<0.001
**Varices% (N)**	7.08% (9)	42.30% (11)	<0.001	4.09% (5)	48.38% (15)	<0.001	3.87% (%)	62.5% (15)	<0.001
**TIPS % (N)**	0.78% (1)	7.69% (2)	0.09	0% (0)	9.67% (3)	0.04	0% (0)	12.5% (3)	0.03
**Malignancy % (N)**	9.44%(12)	30.76% (8)	0.003	8.19% (10)	32.25% (10)	<0.001	9.3% (12)	33.33% (8)	0.001
**All-cause mortality% (N)**	9.44% (12)	26.92% (7)	0.01	6.55% (8)	35.48% (11)	<0.001	5.42% (7)	50% (12)	<0.001

We next performed a multivariate analysis to test the major clinical outcomes in patients with advanced liver fibrosis upon adjustment to gender, age, hypertension and type 2 DM. Since NFS includes impaired fasting glucose or diagnosis of type 2 DM as part of its calculation, type 2 DM was excluded as an adjusted covariate in this specific noninvasive tool, and the adjustment was made only to gender, age and hypertension. Indeed, patients with advanced fibrosis per liver biopsy were more likely to be admitted during the study follow-up (HR 2.07 CI 1.49–2.87, p<0.001) and to have longer hospitalization stays (HR 1.79, CI 1.22–2.63, p = 0.013) also upon adjustment to these variables. Importantly, a multivariate analysis showed that the non-invasive scores APRI, FIB-4 and NFS were all predictors for higher admission rates and longer hospitalizations during the follow-up period, as well (Table 4). We next used a stepwise logistic regression analysis in order to assess the selective contribution of each variable incorporated in the noninvasive scoring systems (S1 Table). Age (OR 1.04, CI 1.01–1.08, p = 0.02) serum platelets (OR 0.98, CI 0.97–0.99, p<0.001) and AST (OR 1.05, CI 1.02–1.08, p<0.001) were significantly associated with higher rates of hospitalizations during follow up.

**Table 4:** Multivariate adjusted hazard ratios and 95% CIs of outcomes by risk score category. The results are adjusted to gender, age, HTN and DM2. The results of NAFLD fibrosis score are adjusted only to gender, age and HTN.

	Fibrosis 3–4			APRI score > 1.5			FIB-4 score > 2.67			NAFLD fibrosis score > 0.676		
	HR	95% C.I.	P value	HR	95% C.I.	P Value	HR	95% C.I.	P Value	HR	95% C.I.	P Value
**Mortality**	5.54	1.817–16.88	0.002	2.85	0.89–9.09	0.07	10.52	2.98–37.07	<0.001	1.58	1.23–1.88	<0.001
**Malignancies**	2.47	0.82–7.38	0.10	4.94	1.92–12.82	<0.001	6.12	2.31–16.17	<0.001	1.27	1.05–1.42	0.008
Liver events*	11.09	4.65–26.45	<0.001	6.55	3.12–13.72	<0.001	13.05	5.78–31.54	<0.001	5.12	2.62–10.01	<0.001
**Admissions**	2.07	1.49–2.87	<0.001	2.49	1.80–3.43	<0.001	3.80	2.79–5.19	<0.001	1.74	1.31–2.31	<0.001
**Duration of hospitalization**	1.79	1.22–2.63	0.01	2.90	2.11–3.98	<0.001	2.69	1.92–3.78	<0.001	1.61	1.23–2.10	<0.001

*Liver events included esophageal varices, hepatic encephalopathy, ascites and TIPS.

### Mortality during patients’ follow-up

19 patients had died during follow-up period. While two patients died as a result of cardiovascular disease, most mortality events resulted from infections (9 deaths) or complication of cirrhosis (8 deaths).

**Fig 1:**
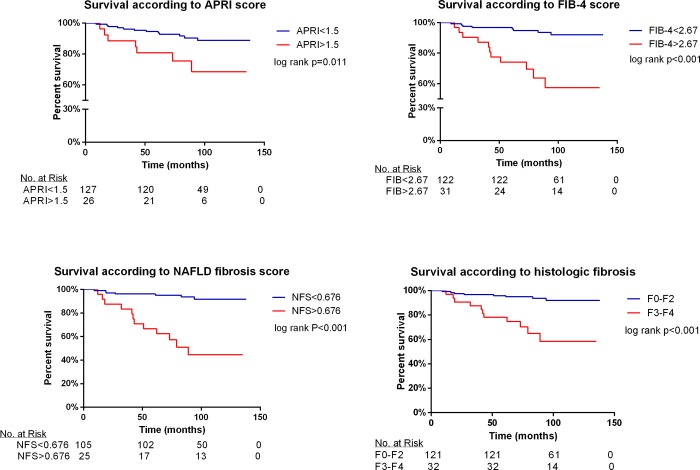
Kaplan-Meier's analysis: survival during the follow-up, according to fibrosis stage, APRI score, FIB-4 score and NAFLD fibrosis score. The number of patients, according to their corresponding scores, in each time point is shown below each graph.

A Kaplan-Meier's analysis clearly shows a significantly better survival during the follow-up period for patients with non-advanced as compared to advanced liver fibrosis, whether determined by liver biopsy or by any one of the three non-invasive tests (Fig 1).

**Fig 2:**
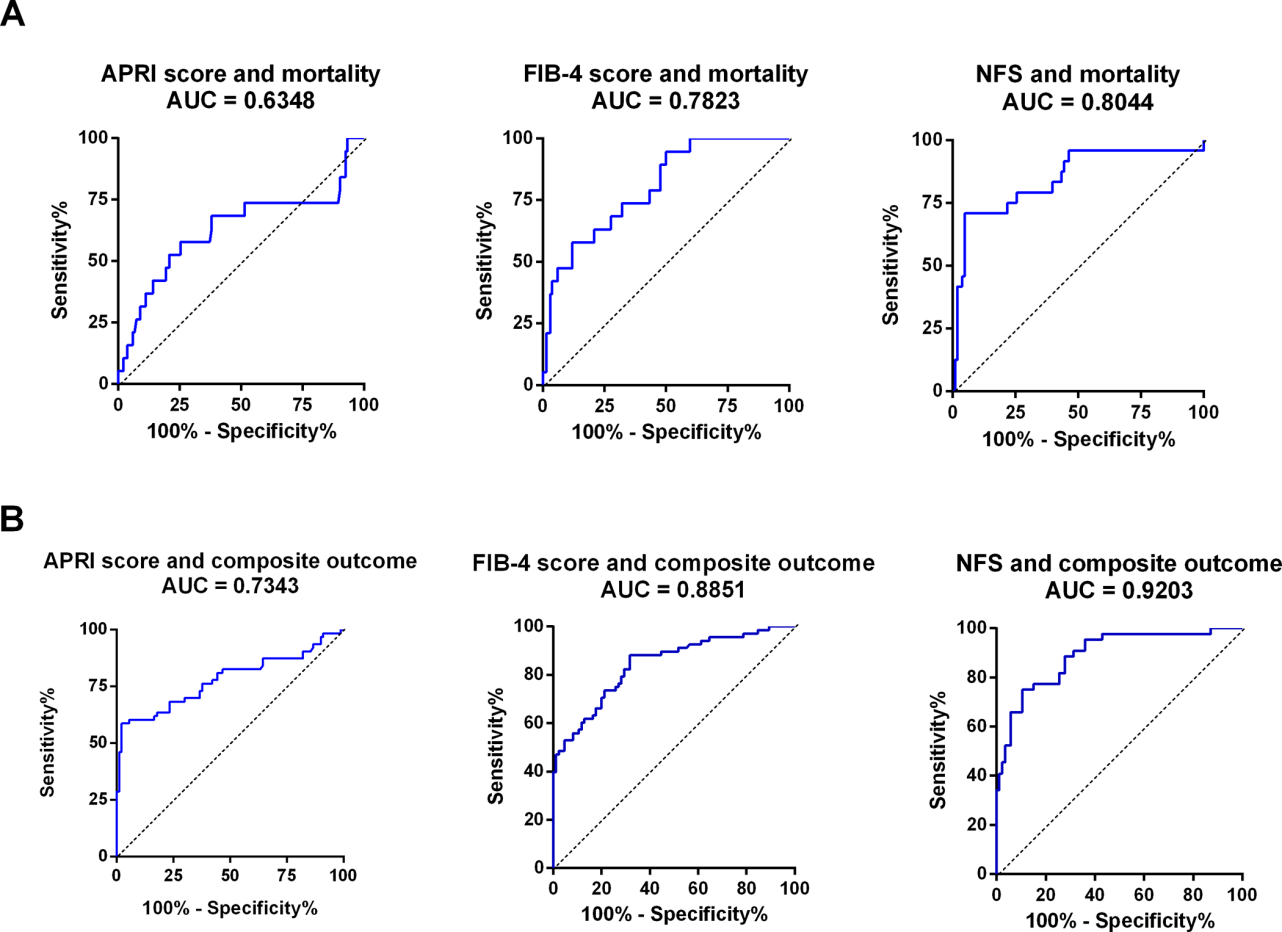
**A**. ROC curve analysis for prediction of mortality according to APRI score > 1.5, FIB-4 score > 2.67 and NAFLD fibrosis score > 0.676. **B**. ROC curve analysis for prediction of composite outcomes of ascites, esophageal varices, hepatic encephalopathy, liver transplantation, TIPS and hospitalizations according to APRI score > 1.5, FIB-4 score > 2.67 and NAFLD fibrosis score (NFS) > 0.676.

The area under the ROC curve (AURC) of the noninvasive scoring systems in prediction mortality is shown in Fig 2A. APRI score higher than 1.5 had the lowest AUC, 0.63, with sensitivity of 54.70% and specificity of 89.43% in prediction mortality. FIB-4 score higher than 2.67 and NFS higher than 0.676 had higher AUCs (0.78 and 0.80, respectively) with sensitivities of 70.11% and 72.40%, respectively, and specificities of 69.08% and 76.25%, respectively, in prediction mortality.

Next, we performed a multivariate analysis to test the performance of non-invasive tests, as compared to liver biopsy, in predicting patients’ mortality. As shown in Table 4, when adjusted to age, gender, hypertension and type 2 DM, liver fibrosis per-biopsy, as well as FIB-4 score and NFS were found to predict mortality during follow-up. In a stepwise logistic regression analysis of the variables included in the noninvasive scoring systems (S2 Table), age (OR 1.06, CI 1.01–1.11, p = 0.02) and serum platelets (OR 0.99, CI 0.98–0.996, p = 0.002) were significantly associated with all-cause mortality during follow up.

### Liver-related events during follow up

The occurrence of ascites, esophageal varices and hepatic encephalopathy during follow up was significantly higher in patients with significant fibrosis stage (Table 2) and higher APRI, FIB-4 and NFS (Table 3). When adjusted to gender, age, hypertension and type 2 DM, the occurrence of liver events was associated with a higher fibrosis stage and higher APRI score, FIB-4 score and NFS (Table 4). The AURCs of the noninvasive scores in predicting patients’ liver-related events and hospitalizations are outlined in Fig 2B. In a stepwise logistic regression analysis (S3 Table) age (OR 1.06, CI 1.04–1.11, p = 0.03) and serum platelets (OR 0.97, CI 0.96–0.98, p<0.001) were significantly associated with the development of liver events during follow up.

### Incidence of malignancies during follow-up

20 patients were diagnosed with cancer during follow up. As expected, the most frequent malignancy was HCC (6 patients) followed by prostate cancer (4), lymphoma (5), non-small cell lung cancer (2), colon adenocarcinoma, pancreas adenocarcinoma and melanoma (1 each). When adjusted to gender, age, hypertension and type 2 DM, the development of malignancies was associated with higher APRI score (HR 4.94, CI 1.92–12.82, p = 0.0009), FIB-4 score (HR 6.12, CI 2.31–16.17, p = 0.0003) and NFS (HR 1.27, CI 1.05–1.42, p = 0.008), but not with advanced histologic fibrosis stage per liver biopsy (HR 2.47, CI 0.82–7.38, p = 0.10), as shown in Table 4. Finally, in a stepwise logistic regression analysis, incorporating the variables included in calculation of the noninvasive scoring system (S4 Table), age (OR 1.07, CI 1.02–1.12, p = 0.007) and serum platelets (OR 0.99, CI 0.98–0.998, p<0.006) were significantly associated with the development of cancer during follow up.

### Association of NASH with liver fibrosis and outcomes

27 patients (17.6% of the study population) had positive features of NASH per liver biopsy, and the presence of NASH was not significantly associated with advanced fibrosis as shown in Table 1. S5 Table describes the baseline characteristics of the study population according to the presence or absence of NASH per liver biopsy. The associations of higher noninvasive scores, as well as major clinical outcomes with NASH are reported in Table 5. NASH was not significantly associated with higher APRI score (p = 0.08), FIB-4 score (p = 0.19) or NFS (p = 0.55). In addition, although NASH was found to be significantly associated with length of hospitalizations and the presence of varices, it was not associated with the development of cancer or all-cause mortality.

**Table 5:** Noninvasive scoring systems and major clinical outcome during the follow up period according to the presence or absence of NASH.

	Negative NASH (N = 126)	Positive NASH (N = 27)	p value
**APRI score > 1.5**	14.28% (18)	29.62% (8)	0.08
**FIB-4 score > 2.67**	18.25% (23)	29.62% (8)	0.19
**NFS > 0.676**	15.07% (19)	18.51% (5)	0.55
**Length of follow up (months) (range)**	97.91 (60.87–144.54)	109.95 (66.93–140.7)	0.01
**Hospitalizations (No.) (range)**	2.11 (0–16)	3.12 (0–15)	0.21
**Length of hospitalizations (days) (range)**	2.10 (0–13)	3.51 (0–18.2)	0.03
**Ascites % (N)**	11.11% (14)	18.51% (5)	0.33
**Encephalopathy % (N)**	7.14% (9)	14.81% (4)	0.19
**Varices% (N)**	10.31% (13)	25.92% (7)	0.03
**TIPS % (N)**	1.58% (2)	3.70% (1)	0.44
**Malignancy % (N)**	13.49% (17)	11.11% (3)	0.73
**All-cause mortality% (N)**	11.11% (14)	18.51% (5)	0.33

APRI = AST to Platelet Ratio Index, NFS = NAFLD fibrosis score, NASH = Nonalcoholic steatohepatitis, TIPS = Trans-jugular Intrahepatic Portosystemic Shunt.

## Discussion

In this single-center, retrospective study of 153 patients with biopsy proven NAFLD, we found that simple noninvasive scores allow identification of patients with NAFLD at a higher risk of liver-related complications, cancer and mortality during an average follow-up of 100 months. Liver biopsy is still considered the gold standard for diagnosing advanced fibrosis, which is the single most important prognostic factor in patients with NAFLD[19]. Our study suggests that non-invasive scores may reliably predict prognosis, including all-cause mortality, when compared to liver biopsy, and may even have an additive value in predicting the future development of cancer in NAFLD patients.

Our study strengthens previous studies that demonstrated the prognostic value of noninvasive scoring systems in patients with NAFLD.[10, 11] In addition, invasive tools such as measurement of hepatic venous pressure gradient (HVPG) were also compared with noninvasive scoring systems and were found to have minor additive values, if at all.[20] Noteworthy, our study is the first to show that significant fibrosis, as assessed by both noninvasive as well as invasive methods, is associated with higher admission rates and longer hospitalization stays. Aside from the economic burden, multiple hospitalizations are implicated in increased risk for health care associated infections[21], which may explain the high rate of infection-related mortality in our study cohort.

Indeed, while previous studies have found cardiovascular related mortality, in addition to liver related mortality, as major causes of death in NAFLD patients[10], our study indicates that infections are a major cause of death in this population, responsible for nearly half of all mortality events. Given that patients with advanced liver disease are in a state of immune dysfunction[22], making them susceptible to life threatening bacterial, among other infections[21], we believe that our observation is compatible with real-life experience of us and others.

Our study shows that noninvasive scoring system, as opposed to actual biopsy-proven advanced liver fibrosis, may herald the future development of cancer. Interestingly, this is true for both liver related as well as to non-liver related cancers. A possible reason for this finding is that noninvasive tools incorporate several clinical and laboratory parameters which, in turn, may more reliably reflect the general health status and conditions that might provide a fertile ground for future emergence of malignancies. Indeed, several studies suggest that elevation in the levels of routinely used liver function tests that are also used in some of the non-invasive tools, such as GGT and ALT, is associated with increased risk for cancer. [23, 24] Interestingly, a series of stepwise regression analyses of the clinical and laboratory parameters which included in the noninvasive scoring systems showed that older age and low platelets were the most important factors associated with our study endpoints.

The most prevalent malignancies in our study cohort were HCC (30%), lymphoma (25%) and prostate cancer (20%). Previous studies have shown that non-cirrhotic patients with NAFLD have over 15-fold higher incidence of HCC, and that among patients with NAFLD those with the higher fibrosis scores had a higher incidence of HCC.[25] Our study revalidates this observation and further expands it to other types of malignancies. In addition, our study shows that assessing liver fibrosis stage by liver biopsy has a limited role in all-cancer related risk stratification, as compared to non-invasive methods, in patients with NAFLD.

NASH, as opposed to advanced fibrosis, was not significantly associated with all-cause mortality or cancer during follow up. Although NASH was found to be associated with longer hospitalizations and the development of esophageal varices, these differences could be attributed to a longer follow up period in NASH patients. Previous studies reported conflicting results regarding the association of NASH with liver and non-liver related mortality and cancer[19, 26], partly due to small number of patients and alteration in NASH definition[27]. Further prospective large scale studies are needed in order to assess the connection between this distinct pathologic appearance and long term outcomes.

In our cohort, gender was not associated with rates of hospitalizations (2.72 in females vs. 1.94 in males, p = 0.19) or length of hospitalization (2.64 days in females vs. 2.11 days in males, p = 0.28), as well as all-cause mortality rates (7.4% in females vs. 16.5% in males, p = 0.14) or liver complications rates (21.2% in females vs. 14.7% in male, p = 0.41). Nonetheless, most malignancy events in our cohort were reported in males (18 events, 90% of the malignancies recorded during follow up, vs. 2 events, 10% of recorded malignancies, in females, p = 0.01). Although several studies already show gender disparities regarding HCC in patients with NAFLD[28], we cannot rule-out that the latter association resulted from the relatively low number of cancer events in our study cohort.

This study has several strengths, including a relatively large patient population with the diagnosis of NAFLD established by liver biopsy for whom detailed and reliable data exists during a long follow-up period. However, this study has several limitations, as well. First, the study is based on patients’ data from only one center of liver diseases. Nevertheless, this center is the largest institute in the country, treating a large population of patients with diverse demographic, socioeconomic, and clinical features and therefore largely represents heterogenic population. Second, as a result of the retrospective nature of this study, the evidence for the development of liver-related events was obtained from the medical electronic records when such events were recorded. It is therefore possible that additional liver-related events have occurred and not fully recorded. Future prospective multicenter studies may address these limitations.

In conclusion, using simple and non-invasive scoring systems in patients with NAFLD may assist in stratifying patients as low or high risk in respect to overall mortality, liver-related complications and recurrent hospitalizations. These scoring systems may even have an advantage over liver biopsy in predicting liver and non-liver related cancer.

## Supporting information

S1 Table A stepwise logistic regression analysis–the association of the parameters included in the noninvasive scoring systems with hospitalizations during follow up. (DOCX)

S2 Table A stepwise logistic regression analysis–the association of the parameters included in the noninvasive scoring systems with all cause mortality during follow up. (DOCX)

S3 Table A stepwise logistic regression analysis–the association of the parameters included in the noninvasive scoring systems with the development of liver events during follow up. (DOCX)

S4 Table A stepwise logistic regression analysis–the association of the parameters included in the noninvasive scoring systems with the development of cancer during follow up. (DOCX)

S5 Table Baseline characteristics of study population according to presence of NASH. (DOCX)

S1 Database A table with the basic epidemiological and laboratory parameters of the patients in this study. (XLSX)
