# Supplementary material for: Noninvasive scoring systems predict hepatic and extra-hepatic cancers in patients with nonalcoholic fatty liver disease
Source: PLoS One. 2018 Aug 14;13(8):e0202393. doi: 10.1371/journal.pone.0202393 (PMC6091950; doi:10.1371/journal.pone.0202393)
Supplement: S3 Table — (DOCX) [file pone.0202393.s003.docx]

**S3 Table. A stepwise logistic regression analysis– the association of the parameters include in the noninvasive scoring systems with the development of liver events during follow up**

|  | **Univariate** | **Multivariate** | | |
| --- | --- | --- | --- | --- |
|  | **p value** | **p value** | **OR** | **95% CI** |
| **Age** | **0.003** | **0.03** | **1.06** | **1.04 – 1.11** |
| **BMI** | 0.92 |  |  |  |
| **Positive DM2 diagnosis** | 0.16 |  |  |  |
| **Platelets** | **<0.001** | **<0.001** | **0.97** | **0.96 – 0.98** |
| **Albumin** | **<0.001** |  |  |  |
| **AST** | 0.83 |  |  |  |
| **ALT** | **0.01** |  |  |  |
