# Supplementary material for: Noninvasive scoring systems predict hepatic and extra-hepatic cancers in patients with nonalcoholic fatty liver disease
Source: PLoS One. 2018 Aug 14;13(8):e0202393. doi: 10.1371/journal.pone.0202393 (PMC6091950; doi:10.1371/journal.pone.0202393)
Supplement: S4 Table — (DOCX) [file pone.0202393.s004.docx]

**S4 Table. A stepwise logistic regression analysis– the association of the parameters include in the noninvasive scoring systems with the development of cancer during follow up**

|  | **Univariate** | **Multivariate** | | |
| --- | --- | --- | --- | --- |
|  | **p value** | **p value** | **OR** | **95% CI** |
| **Age** | **0.003** | **0.007** | **1.07** | **1.02 – 1.12** |
| **BMI** | **0.05** |  |  |  |
| **Positive DM2 diagnosis** | 0.87 |  |  |  |
| **Platelets** | **0.003** | **0.006** | **0.99** | **0.98 – 0.998** |
| **Albumin** | 0.16 |  |  |  |
| **AST** | 0.39 |  |  |  |
| **ALT** | 0.35 |  |  |  |
