# Supplementary material for: Noninvasive scoring systems predict hepatic and extra-hepatic cancers in patients with nonalcoholic fatty liver disease
Source: PLoS One. 2018 Aug 14;13(8):e0202393. doi: 10.1371/journal.pone.0202393 (PMC6091950; doi:10.1371/journal.pone.0202393)
Supplement: S5 Table — (DOCX) [file pone.0202393.s005.docx]

**S5 Table. Baseline characteristics of study population according to presence of NASH.**

|  | **Negative NASH (N=126)** | **Positive NASH (N=27)** | **p value** |
| --- | --- | --- | --- |
| **Gender–male%(N)** | 57.14% (72) | 48.14% (13) | 0.39 |
| **Age (range)** | 49.22 (20 – 78) | 50.52 (30 – 76) | 0.63 |
| **BMI (range)** | 29.33 (17.48 – 43.85) | 30.03 (22.00 – 37.85) | 0.44 |
| **Statin use % (N)** | 53.17% (67) | 59.25% (16) | 0.56 |
| **HTN diagnosis% (N)** | 39.68% (50) | 48.14% (13) | 0.52 |
| **DM2 % (N)** | 61.90% (78) | 70.37% (19) | 0.40 |
| **Hemoglobin A1C (%) (range)** | 6.23 (4.60 – 14.00) | 6.56 (4.50 – 11.90) | 0.31 |
| **Total cholesterol (mg/dL) (range)** | 178.57 (69 – 293) | 173.78 (102 – 321) | 0.56 |
| **Triglycerides (mg/dL) (range)** | 162.95 (55- 578) | 154.48 (45 – 430) | 0.66 |
| **Creatinine (mg/dL) (range)** | 0.79 (0.39 – 2.23) | 0.74 (0.29 – 1.03) | 0.31 |
| **INR (range)** | 1.07 (0.85 – 1.15) | 1.08 (0.90 – 1.40) | 0.81 |
| **Albumin (g/dL) (range)** | 4.21 (1.90 – 5.30) | 3.98 (2.00 – 4.70) | 0.08 |
| **Platelets (range)** | 225.56 (32 – 390) | 196.11 (32 – 370) | 0.09 |
| **AST (U/L) (range)** | 54.83 (12 – 212) | 66.33 (11 – 192) | 0.17 |
| **ALT (U/L) (range)** | 71.79 (9 – 323) | 83.04 (11 – 229) | 0.29 |
| **Steatosis grade** |  |  | 0.85 |
| **Steatosis grade 1 % (N)** | 38.88% (49) | 37.03% (10) |  |
| **Steatosis grade 2 % (N)** | 42.85% (54) | 48.14% (13) |  |
| **Steatosis grade 3 % (N)** | 18.25% (23) | 14.81% (4) |  |
| **APRI Score (range)** | 1.02 (0.17 – 8.94) | 1.34 (0.09 – 4.42) | 0.22 |
| **FIB-4 Score (range)** | 2.16 (0.34 – 29.36) | 2.64 (0.27 – 9.98) | 0.48 |
| **NFS (range)** | -1.08 (-4.85 – 4.69) | -0.28 (-4.22 – 3.63) | 0.08 |
| **Presence of F3-F4** | 18.25% (23) | 33.33% (9) | 0.11 |

NFS = NAFLD fibrosis score, HTN = hypertension, DM2 = type 2 diabetes mellitus, APRI= AST to Platelet Ratio Index, NASH = Nonalcoholic steatohepatitis
